# Large-scale directed network inference with multivariate transfer entropy and hierarchical statistical testing

**DOI:** 10.1162/netn_a_00092

**Published:** 2019-07-01

**Authors:** Leonardo Novelli, Patricia Wollstadt, Pedro Mediano, Michael Wibral, Joseph T. Lizier

**Affiliations:** Centre for Complex Systems, Faculty of Engineering, The University of Sydney, Sydney, Australia; Honda Research Institute Europe, Offenbach am Main, Germany; Computational Neurodynamics Group, Department of Computing, Imperial College London, London, United Kingdom; Campus Institute for Dynamics of Biological Networks, Georg-August University, Göttingen, Germany; Centre for Complex Systems, Faculty of Engineering, The University of Sydney, Sydney, Australia

**Keywords:** Neuroimaging, Directed connectivity, Effective network, Multivariate transfer entropy, Information theory, Nonlinear dynamics, Statistical inference, Nonparametric tests

## Abstract

Network inference algorithms are valuable tools for the study of large-scale neuroimaging datasets. Multivariate transfer entropy is well suited for this task, being a model-free measure that captures nonlinear and lagged dependencies between time series to infer a minimal directed network model. Greedy algorithms have been proposed to efficiently deal with high-dimensional datasets while avoiding redundant inferences and capturing synergistic effects. However, multiple statistical comparisons may inflate the false positive rate and are computationally demanding, which limited the size of previous validation studies. The algorithm we present—as implemented in the IDTxl open-source software—addresses these challenges by employing hierarchical statistical tests to control the family-wise error rate and to allow for efficient parallelization. The method was validated on synthetic datasets involving random networks of increasing size (up to 100 nodes), for both linear and nonlinear dynamics. The performance increased with the length of the time series, reaching consistently high precision, recall, and specificity (>98% on average) for 10,000 time samples. Varying the statistical significance threshold showed a more favorable precision-recall trade-off for longer time series. Both the network size and the sample size are one order of magnitude larger than previously demonstrated, showing feasibility for typical EEG and magnetoencephalography experiments.

## INTRODUCTION

The increasing availability of large-scale, fine-grained datasets provides an unprecedented opportunity for quantitative studies of complex systems. Nonetheless, a shift toward data-driven modeling of these systems requires efficient algorithms for analyzing multivariate time series, which are obtained from observation of the activity of a large number of elements.

In the field of neuroscience, the multivariate time series typically obtained from brain recordings serve to infer minimal (effective) network models which can explain the dynamics of the nodes in a neural system. The motivation for such models can be, for instance, to describe a causal network (Ay & Polani, 2008; Friston, 1994) or to model the directed information flow in the system (Vicente et al., 2011) in order to produce a minimal computationally equivalent network (Lizier & Rubinov, 2012).

Information theory (Cover & Thomas, 2005; Shannon, 1948) is well suited for the latter motivation of inferring networks that describe information flow as it provides model-free measures that can be applied at different scales and to different types of recordings. These measures, including conditional mutual information (Cover & Thomas, 2005) and transfer entropy (Schreiber, 2000), are based purely on probability distributions and are able to identify nonlinear relationships (Paluš et al., 1993). Most importantly, information-theoretic measures allow the interpretation of the results from a distributed computation or information processing perspective, by modeling the information storage, transfer, and modification within the system (Lizier, 2013). Therefore, information theory simultaneously provides the tools for building the network model and the mathematical framework for its interpretation.

The general approach to network model construction can be outlined as follows: for any *target* process (element) in the system, the inference algorithm selects the *minimal set* of processes that collectively contribute to the computation of the target’s next state. Every process can be separately studied as a target, and the results can be combined into a directed network describing the information flows in the system. This task presents several challenges:

•  The state space of the possible network models grows faster than exponentially with respect to the size of the network;
•  Information-theoretic estimators suffer from the “curse of dimensionality” for large sets of variables (Paninski, 2003; Roulston, 1999);
•  In a network setting, statistical significance testing requires multiple comparisons. This results in a high false positive rate (type I errors) without adequate family-wise error rate controls (Dickhaus, 2014) or a high false negative rate (type II errors) with naive control procedures;
•  Nonparametric statistical testing based on shuffled surrogate time series is computationally demanding but currently necessary when using general information-theoretic estimators (Bossomaier et al., 2016; Lindner et al., 2011).

Several previous studies (Faes et al., 2011; Lizier & Rubinov, 2012; Sun et al., 2015; Vlachos & Kugiumtzis, 2010) proposed greedy algorithms to tackle the first two challenges outlined above (see a summary by Bossomaier et al., 2016, sec 7.2). These algorithms mitigate the curse of dimensionality by greedily selecting the random variables that iteratively reduce the uncertainty about the present state of the target. The reduction of uncertainty is rigorously quantified by the information-theoretic measure of conditional mutual information (CMI), which can also be interpreted as a measure of conditional independence (Cover & Thomas, 2005). In particular, these previous studies employed multivariate forms of the transfer entropy, that is, conditional and collective forms (Lizier et al., 2008, 2010). In general, such greedy optimization algorithms provide a locally optimal solution to the NP-hard problem of selecting the most informative set of random variables. An alternative optimization strategy—also based on conditional independence—employs a preliminary step to prune the set of sources (Runge et al., 2012, 2018). Despite this progress, the computational challenges posed by the estimation of multivariate transfer entropy have severely limited the size of problems investigated in previous validation studies in the general case of nonlinear estimators, for example, Montalto et al. (2014) used 5 nodes and 512 samples; Kim et al. (2016) used 6 nodes and 100 samples; Runge et al. (2018) used 10 nodes and 500 samples. However, modern neural recordings often provide hundreds of nodes and tens of thousands of samples.

These computational challenges, as well as the multiple testing challenges described above, are addressed here by the implementation of rigorous statistical tests, which represent the main theoretical contribution of this paper. These tests are used to control the family-wise error rate and are compatible with parallel processing, allowing the simultaneous analysis of the targets. This is a crucial feature, which enabled an improvement on the previous greedy algorithms. Exploiting the parallel computing capabilities of high-performance computing clusters and graphics processing units (GPUs) enabled the analysis of networks at a relevant scale for brain recordings—up to 100 nodes and 10,000 samples. Our algorithm has been implemented in the recently released IDTxl Python package (the “Information Dynamics Toolkit xl”; Wollstadt et al., 2019).

We validated our method on synthetic datasets involving random structural networks of increasing size (also referred to as *ground truth*) and different types of dynamics (vector autoregressive processes and coupled logistic maps). In general, effective networks are able to reflect dynamic changes in the regime of the system and do not reflect an underlying structural network. Nonetheless, in the absence of hidden nodes (and other assumptions, including stationarity and the causal Markov condition), the inferred information network was proven to reflect the underlying structure for a sufficiently large sample size (Sun et al., 2015). Experiments under these conditions provide arguably the most important validation that the algorithm performs as expected, and here we perform the first large-scale empirical validation for non-Gaussian variables. As shown in the Results, the performance of our algorithm increased with the length of the time series, reaching consistently high precision, recall, and specificity ( >98% on average) for 10,000 time samples. Varying the statistical significance threshold showed a more favorable precision-recall trade-off for longer time series.

## METHODS

### Definitions and assumptions

**Figure 1.**
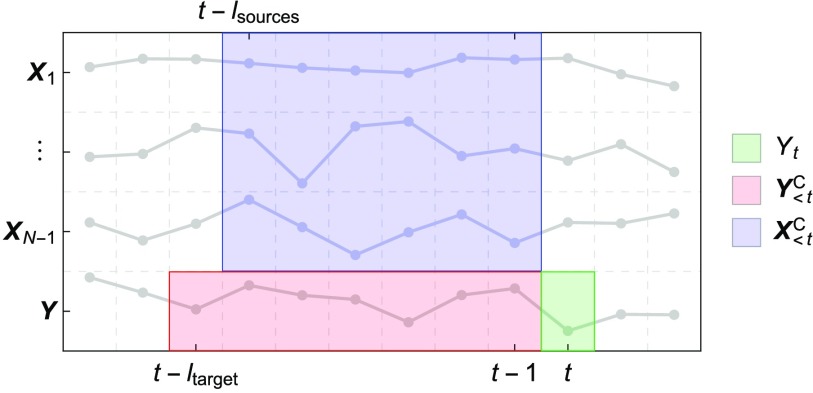
Example of a possible definition of the candidate sets. The bottom row represents the time series of the target process ***Y***, with the present state *Y*_*t*_ highlighted in green and the candidate target past set $Y_{<t}^{C}$ highlighted in red (up to a lag *l*_target_). The remaining rows represent the time series of the source processes ***X***_*i*_, with the candidate sources past set $X_{<t}^{C}$ highlighted in blue (up to a lag *l*_sources_). For simplicity, only a single trial of the experiment is represented.

**Figure 2.**
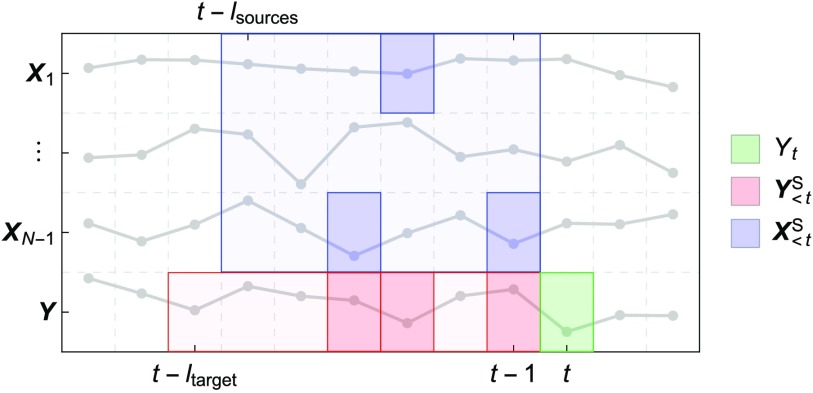
Example of a resulting nonuniform embedding of the time series relevant to *Y*_*t*_. The bottom row represents the time series of the target process ***Y***, with the present state *Y*_*t*_ highlighted in green and the selected target past set $Y_{<t}^{S}$ highlighted in red (as a subset of the candidate target past set shown in light red). The remaining rows represent the time series of the source processes ***X***_*i*_, with the selected sources past set $X_{<t}^{S}$ highlighted in blue (as a subset of the candidate sources past set shown in light blue). The embedding only specifies the *relative* lags between the variables. For simplicity, only a single trial of the experiment is shown.

Let us consider a system of *N* discrete-time stochastic processes for which a finite number of samples have been recorded (over time and/or in different replications of the same experiment). In general, let us assume that the stochastic processes are stationary in each experimental time-window and Markovian with finite memory *l*_M_. Further assumptions will be made for the validation study. The following quantities are needed for the setup and formal treatment of the algorithm and are visualized in Figure 1 and Figure 2:

**Target process*Y***: a process of interest within the system (where ***Y*** = {*Y*_*t*_ ∣ *t* ∈ ℕ}); the choice of the target process is arbitrary and all the processes in the system can separately be studied as targets.
**Source processes*X***_*i*_: the remaining processes within the system (where *i* = 1, …, *N* − 1 and ***X***_*i*_ = {*X*_*i*,*t*_ ∣ *t* ∈ ℕ}).
**Sample number (or size)***T*: the number of samples recorded over time.
**Replication number***R*: the number of replications of the same experiment (e.g., trials).
**Target present state***Y*_*t*_: the random variable (RV) representing the state of the target at time *t* (where *t* ≤ *T*), whose information contributors will be inferred.
**Candidate target past**$Y_{<t}^{C}$: an arbitrary finite set of RVs in the past of the target, up to a maximum lag *l*_target_, i.e., $Y_{<t}^{C}$ = {*Y*_*t*−1_, …, *Y*_*t*−*l*_target__}.
**Candidate sources past**$X_{<t}^{C}$: an arbitrary finite set of RVs in the past of the sources, up to a maximum lag *l*_sources_, i.e., $X_{<t}^{C}$ = {*X*_*i*,*t*−1_, …, *X*_*i*,*t*−*l*_sources__ ∣ *i* = 1, …, *N* − 1}.
**Selected target past**$Y_{<t}^{S}$: the subset of RVs within the candidate target past set $Y_{<t}^{C}$ that maximally reduces the uncertainty about the present state of the target.
**Selected sources past**$X_{<t}^{S}$: the subset of RVs within the candidate sources past set $X_{<t}^{C}$ that maximally further reduces the uncertainty about the present state of the target, in the context of the selected target past (explained in detail in the following section).

### Inference Algorithm

For a given target process ***Y***, the goal of the algorithm is to infer the minimal set of information contributors to *Y*_*t*_—defined as the selected sources past $X_{<t}^{S}$—in the context of the relevant information contributors from the candidate target past set, defined as the selected target past $Y_{<t}^{S}$.

The algorithm operates in four steps:

1.  Select variables in the candidate target past set $Y_{<t}^{C}$ to obtain $Y_{<t}^{S}$.
2.  Select variables in the candidate sources past set $X_{<t}^{C}$ to obtain $X_{<t}^{S}$.
3.  Prune the selected sources past variables.
4.  Test relevant variables collectively for statistical significance.

The operations performed in the four steps are described in detail hereafter; the result is a *nonuniform embedding* of the target and sources time series (Faes et al., 2011; Takens, 1981; Vlachos and Kugiumtzis, 2010), as illustrated in Figure 2.

#### Step 1: Select variables in the candidate target past set.

The goal of the first step is to find the subset of RVs within the candidate target past set $Y_{<t}^{C}$ that maximally reduces the uncertainty about the present state of the target while meeting statistical significance requirements. Let $Y_{<t}^{S}$ be the *selected target past* set found via optimization under these criteria.

Finding the globally optimal embedding is an NP-hard problem and requires testing all the subsets of the candidate target past set. Since the number of subsets grow exponentially with the size of the candidate set, this is computationally unfeasible; therefore, a greedy approximation algorithm is employed to find a locally optimal solution in the search space of possible embeddings. This approach tackles the challenge of computational complexity by aiming at identifying a minimal conditioning set; in doing so, it also tackles the curse of dimensionality in the estimation of information-theoretic functionals.

The set $Y_{<t}^{S}$ is initialized as an empty set and it is iteratively built up via the following algorithm:

a.  For each candidate variable *C* ∈ $Y_{<t}^{C}$, estimate the CMI contribution *I*(*C*; *Y*_*t*_|$Y_{<t}^{S}$);
b.  Find the candidate C* which maximizes the CMI contribution (reduction of uncertainty) and perform a statistical significance test against the null hypothesis of conditional independence, that is, that the new variable does not further reduce the uncertainty in the context of the previously included variables. If significant, add C* to $Y_{<t}^{S}$ and remove it from $Y_{<t}^{C}$. The *maximum statistic* is employed to control the family-wise error rate (explained in detail in the *Statistical Tests* section);
c.  Repeat the previous steps until the maximum CMI contribution is not significant or $Y_{<t}^{C}$ is empty.

From a distributed, intrinsic computation perspective, the goal can be interpreted as finding the embedding of the target’s past states that maximizes the *active information storage* (Lizier et al., 2012) to ensure self-prediction optimality as suggested by Wibral et al. (2013). This approach is similar to the one proposed by Garland et al. (2016) but uses nonuniform embedding and additional statistical controls.

The nonuniform embedding of the time series was introduced by Vlachos and Kugiumtzis (2010) and Faes et al. (2011), who used an arbitrary threshold for the conditional mutual information. Lizier and Rubinov (2012) introduced a statistical significance test to select the candidates, which this study builds on in proposing the maximum statistic. In addition, they embedded the target time series before embedding the sources, that is, the active information storage is modeled first and the information transfer is then examined in that context, thereby taking a specific modeling perspective on the information processing carried out by the system.

#### Step 2: Select variables in the candidate sources past set.

The goal of the second step is to find the subset of RVs within the candidate sources past set $X_{<t}^{C}$ that maximally further reduces the uncertainty about the present state of the target, in the context of the selected target past found in the first step. Let $X_{<t}^{S}$ be the *selected sources past* set found via optimization under these criteria.

As for step 1, a greedy approximation algorithm is employed, and the statistical significance is tested throughout the selection process. $X_{<t}^{S}$ is initialized as an empty set and it is iteratively built up via the following algorithm:

a.  For each candidate variable *C* ∈ $X_{<t}^{C}$, estimate the conditional transfer entropy contribution *I*(*C*; *Y*_*t*_|$Y_{<t}^{S}$, $X_{<t}^{S}$) (Lizier et al., 2008, 2010; Vakorin et al., 2009; Verdes, 2005). When $X_{<t}^{S}$ is empty, this is simply a pairwise or bivariate transfer entropy (Schreiber, 2000); using the conditional form serves to prevent candidates carrying only redundant information (due to, e.g., common driver or pathway effects) from being selected, as well as to capture synergistic interactions between *C* and $X_{<t}^{S}$.
b.  Find the candidate C* which maximizes the conditional transfer entropy contribution (reduction of uncertainty) and perform a statistical significance test against the null hypothesis of conditional independence: if significant, add C* to $X_{<t}^{S}$ and remove it from $X_{<t}^{C}$. The *maximum statistic* is employed to control the family-wise error rate.
c.  Repeat the previous steps until the maximum conditional transfer entropy contribution is not significant or $X_{<t}^{C}$ is empty.

From a distributed computation perspective, the goal can be interpreted as finding the nonuniform embedding of the source processes’ past that maximizes the *collective transfer entropy* to the target, defined as *I*($X_{<t}^{S}$; *Y*_*t*_|$Y_{<t}^{S}$) (Lizier et al., 2010). As above, the rationale for embedding the past of the sources as a second step is to achieve optimal separation of the storage and transfer contributions (Lizier & Rubinov, 2012).

#### Step 3: Prune the selected sources past variables.

The third step of the algorithm is a pruning procedure performed to ensure that the variables included in the early iterations of the second step still provide a statistically significant information contribution in the context of the final selected sources past set $X_{<t}^{S}$. The pruning step involves the following operations:

a.  For each variable *C* ∈ $X_{<t}^{S}$, estimate the conditional mutual information contribution *I*(*C*; *Y*_*t*_|$Y_{<t}^{S}$, $X_{<t}^{S}$ ∖ {*C*}), where the set difference operation is performed to exclude the variable *C* from the conditioning set;
b.  Find the variable C* which minimizes the CMI contribution and perform a statistical significance test: if *not* significant, remove *C* from $X_{<t}^{S}$. The *minimum statistic* is employed to test for significance against the null hypothesis of conditional independence while controlling the family-wise error rate;
c.  Repeat the previous steps until the minimum CMI contribution is not significant or $X_{<t}^{S}$ is empty.

The pruning step was introduced by Lizier & Rubinov (2012); remarkably, Sun et al. (2015) proved that this step is essential for the theoretical convergence of the inferred network to the causal network in the Granger-Wiener framework; they also rigorously laid out the mathematical assumptions needed for such convergence (see *Validation Tasks* section).

#### Step 4: Test relevant variables collectively for statistical significance.

The fourth and final step of the algorithm is the computation of the collective transfer entropy from the selected sources past set $X_{<t}^{S}$ to the target and the performance of an *omnibus test* to ensure statistical significance against the null hypothesis of conditional independence. The resulting omnibus *p* value can further be used for correction of the family-wise error rate if the inference is carried out for multiple targets. The set $X_{<t}^{S}$ is only accepted as a result if all the statistical tests are passed. Importantly, the selected sources set $X_{<t}^{S}$, inferred in the context of $Y_{<t}^{S}$, is the final result of the algorithm for a given target process ***Y***. The order in which variables were inferred is not relevant.

The statistical tests play a fundamental role in the inference and provide the stopping conditions for the iterations involved in the first and second steps of the algorithm. These stopping conditions are adaptive and change according to the amount of data available (the length of the time series). Given their importance, the statistical tests are described in detail in the following section.

### Statistical Tests

The crucial steps in the inference algorithm rely on determining whether the CMI is positive. However, due to the finite sample size, the CMI estimators may produce nonzero estimates in the case of zero CMI, and it may even return negative estimates if the estimator bias is larger than the true CMI (Kraskov et al., 2004; Roulston, 1999). For this reason, statistical tests are required to assess the significance of the CMI estimates against the null hypothesis of no CMI (i.e., conditional independence) (Chávez et al., 2003; Lindner et al., 2011; Lizier et al., 2011; Vicente et al., 2011).

For certain estimators, analytic solutions exist for the finite-sample distribution under this null hypothesis (see Lizier, 2014); in the absence of an analytic solution, the null distributions are computed in a nonparametric way by using surrogate time series (Schreiber & Schmitz, 2000). The surrogates are generated to satisfy the null hypothesis by destroying the temporal relationship between the source and the target while preserving the temporal dependencies within the sources.

Finally, the inference algorithm is based on multiple comparisons and requires an appropriate calibration of the statistical tests to achieve the desired family-wise error rate (i.e., the probability of making one or more false discoveries, or *type I errors*, when performing multiple hypotheses tests). The maximum statistic and minimum statistic tests employed in this study were specifically conceived to tackle these challenges.

#### Maximum statistic test.

The maximum statistic test is a step-down statistical test used to control the family-wise error rate when selecting the past variables for the target and source embeddings, which involves multiple comparisons.

Let us first consider the first step of the main algorithm and assume that we have picked the single candidate variable C* (from the candidate target past set $Y_{<t}^{C}$), which maximizes the CMI contribution. The maximum statistic test mirrors this selection process by picking the maximum value among the surrogates. Specifically, let I* := *I*(C*; *Y*_*t*_|$Y_{<t}^{S}$) be the maximum contribution (i.e., the maximum statistic); the following algorithm is used to test I* for statistical significance:

1.  For each *C*_*j*_ ∈ $Y_{<t}^{C}$, generate *S* surrogates time series $C_{j,1}^{\prime }$, …, $C_{j,S}^{\prime }$ and compute the corresponding surrogate CMI values $I_{j,1}^{\prime }$ = *I*($C_{j,1}^{\prime }$; *Y*_*t*_|$Y_{<t}^{S}$), …, $I_{j,S}^{\prime }$ = *I*($C_{j,S}^{\prime }$; *Y*_*t*_|$Y_{<t}^{S}$). More details about the surrogate generation are provided at the end of this section. The number of surrogates *S* must be chosen according to the desired significance level *α*_max_, i.e., such that *S* > 1/*α*_max_.
2.  Compute the maximum CMI value over candidates $I_{s}^{*}$ := *max*($I_{1,s}^{\prime }$, …, $I_{n,s}^{\prime }$) for each surrogate *s* = 1, …, *S*. Here, *n* denotes the number of candidates and hence the number of comparisons. The obtained values $I_{1}^{*}$, …, $I_{S}^{*}$ provide the (empirical) null distribution of the maximum statistic (see Table 1).
3.  Calculate the *p* value for I* as the fraction of surrogate maximum statistic values that are larger than I*.
4.  I* is deemed *significant* if the *p* value is smaller than *α*_max_ (i.e., the null hypothesis of conditional independence for the candidate variable with the maximum CMI contribution is rejected at level *α*_max_).

**Table 1.** Computing the null distribution of the maximum statistic. The null distribution is empirically described by the values $I_{1}^{*}$, …, $I_{S}^{*}$, obtained as $I_{s}^{*}$ := *max*($I_{1,s}^{\prime }$, …, $I_{n,s}^{\prime }$), for each surrogate *s* = 1, …, *S*. Here, *n* denotes the number of candidates and hence the number of comparisons. The null distribution is used to test the significance of I* against the null hypothesis of zero CMI.

	Variable *C*_*j*_ ∈ $Y_{<t}^{C}$	CMI *I*_*j*_ = *I*(*C*_*j*_; *Y*_*t*_|$Y_{<t}^{S}$)	Surrogate variables				Surrogate CMI			
			1	2	⋯	*S*	1	2	⋯	*S*
	*C*_1_	*I*_1_	$C_{1,1}^{\prime }$	$C_{1,2}^{\prime }$	⋯	$C_{1,S}^{\prime }$	$I_{1,1}^{\prime }$	$I_{1,2}^{\prime }$	⋯	$I_{1,S}^{\prime }$
	*C*_2_	*I*_2_	$C_{2,1}^{\prime }$	$C_{2,2}^{\prime }$	⋯	$C_{2,S}^{\prime }$	$I_{2,1}^{\prime }$	$I_{2,2}^{\prime }$	⋯	$I_{2,S}^{\prime }$
	⋮	⋮	⋮	⋮		⋮	⋮	⋮		⋮
	*C*_*n*_	*I*_*n*_	$C_{n,1}^{\prime }$	$C_{n,2}^{\prime }$	⋯	$C_{n,S}^{\prime }$	$I_{n,1}^{\prime }$	$I_{n,2}^{\prime }$	⋯	$I_{n,S}^{\prime }$
max CMI		I*					$I_{1}^{*}$	$I_{2}^{*}$	⋯	$I_{S}^{*}$

The variables and quantities used in the above algorithm are presented in Table 1. The key goal in the surrogate generation is to preserve the temporal order of samples in the target time series *Y*_*t*_ (which is not shuffled) and preserve the distribution of the sources *C*_*j*_ while destroying any potential relationships between the sources and the target (Vicente et al., 2011). This can be achieved in multiple ways. If multiple replications (e.g., trials) are available, surrogate data is generated by shuffling the order of replications for the candidate *C*_*j*_ while keeping the order of replications for the remaining variables intact. When the number of replications is not sufficient to guarantee enough permutations, the embedded source samples within individual trials are shuffled instead (see Chávez et al., 2003; Lizier et al., 2011; Verdes, 2005; Vicente et al., 2011; and the summary by Lizier, 2014, Appendix A.5). Note that the generation of surrogates (steps 1-3) can be avoided when the null distributions can be derived analytically, for example, with Gaussian estimators (Barnett & Bossomaier, 2012).

The same test is performed during the selection of the variables in the candidate sources past set (step 2 of the main algorithm), with the only difference that *C*_*j*_ ∈ $X_{<t}^{C}$ and that $X_{<t}^{S}$ is added to the conditioning set, that is, $I_{j,s}^{\prime }$ = *I*($C_{j,s}^{\prime }$; *Y*_*t*_|$Y_{<t}^{S}$, $X_{<t}^{S}$) for each surrogate *s* = 1, …, *S*.

#### Family-wise error rate correction.

How does the maximum statistic test control the family-wise error rate? Intuitively, one or more statistics will exceed a given threshold if and only if the maximum exceeds it. This relationship can be used to obtain an adjusted threshold from the distribution of the maximum statistic under the null hypothesis, which can be used to control the family-wise error rate both in the weak and strong sense (Nichols & Hayasaka, 2003).

Let us quantify the false positive rate *v*_FPR_ for a single variable when the maximum statistic at the significance level *α*_max_ is employed. For simplicity, the derivation is performed under the hypothesis that the information contributors to the target have been selected in the first iterations of the greedy algorithm and removed from the candidate sources past set $X_{<t}^{C}$. Under this hypothesis, the target is conditionally independent of the remaining *n* variables in $X_{<t}^{C}$ given the selected source and target variables. Let *I*_1_, …, *I*_*n*_ be the corresponding CMI estimates and let *I*_max_ := max(*I*_1_, …, *I*_*n*_) be the maximum statistic. As discussed above, the estimates might be positive even under the conditional independence hypothesis, due to finite-sample effects. Since the estimates are independently obtained from shuffled time series, they are treated as i.i.d. RVs.

Let *i*_threshold_ be the critical threshold corresponding to the given significance value *α*_max_, that is, *i*_threshold_ := sup{*x* ∈ ℝ|*P*(*I*_max_ ≥ *x*) = *α*_max_}. Then

$$\begin{array}{ccc} \alpha_{\text{max}} & = & P\left(I_{\text{max}}>i_{\text{threshold}}\right)\\ & = & 1-P\left(I_{1}\leq i_{\text{threshold}},\ldots ,I_{n}\leq i_{\text{threshold}}\right)\\ & = & 1-\overset{n}{\underset{j=1}{\prod }}P\left(I_{j}\leq i_{\text{threshold}}\right)\\ & = & 1-P{\left(I_{1}\leq i_{\text{threshold}}\right)}^{n}\\ & = & 1-{\left(1-v_{\text{FPR}}\right)}^{n} \end{array}$$ (1)

Therefore,

$$v_{\text{FPR}}=1-{\left(1-\alpha_{\text{max}}\right)}^{1/n}$$ (2)

Interestingly, Equation 2 shows that the maximum statistic correction is equivalent to the Dunn-Šidák correction (Šidák, 1967). Performing a Taylor expansion of Equation 2 around *α*_max_ = 0 yields:

$$v_{\text{FPR}}=\overset{\infty }{\underset{j=1}{\sum }}\frac{-\overset{j-1}{\underset{k=0}{\prod }}(kn-1)}{j!}{\left(\frac{\alpha_{\text{target}}}{n}\right)}^{j}$$ (3)

Truncating the Taylor series at *j* = 1 yields the first-order approximation

$$v_{\text{FPR}}\approx \frac{\alpha_{\text{max}}}{n},$$ (4)

which coincides with the false positive rate resulting from the Bonferroni correction (Dickhaus, 2014). Moreover, since the summands in Equation 3 are positive for every *j*, the Taylor series is lower bounded by any truncated series. In particular, the false positive rate resulting from the Bonferroni correction is a lower bound for the *v*_FPR_ (the false positive rate for a single variable resulting from the maximum statistic test), that is, the maximum statistic correction is less stringent than the Bonferroni correction.

Let us now study the effect of the maximum statistic test on the family-wise error rate *t*_FPR_ for a single target while accounting for all the iterations performed during the step-down test, (i.e., *t*_FPR_ is the probability that at least one of the selected sources is a false positive). We have:

$$\begin{array}{ccc} t_{\text{FPR}} & = & \overset{n}{\underset{j=1}{\sum }}P(\text{“the source selected on step j is false positive”})\\ & = & \overset{n}{\underset{j=1}{\sum }}\alpha_{\text{max}}^{j}=\alpha_{\text{max}}\left(\frac{1-\alpha_{\text{max}}^{n}}{1-\alpha_{\text{max}}}\right) \end{array}$$ (5)

Therefore,

$$t_{\text{FPR}}\approx \alpha_{\text{max}}$$ (6)

for the typical small values of *α*_max_ used in statistical testing (even in the limit of large *n*), which shows that *α*_max_ effectively controls the family-wise error rate for a single target.

#### Minimum statistic test.

The minimum statistic test is employed during the third main step of the algorithm (pruning step) to remove the selected variables that have become redundant in the context of the final set of selected source past variables $X_{<t}^{S}$, while controlling the family-wise error rate. This is necessary because of the multiple comparisons involved in the pruning procedure. The minimum statistic test works identically to the maximum statistic test (replacing “maximum” with “minimum” in the algorithm presented above).

#### Omnibus test.

Let T* := *I*($X_{<t}^{S}$; *Y*_*t*_|$Y_{<t}^{S}$) be the collective transfer entropy from all the selected sources past variables $X_{<t}^{S}$ to the target ***Y***. The value T* is tested for statistical significance against the null hypothesis of zero transfer entropy (this test is referred to as the omnibus test). The null distribution is built using surrogates time series obtained via shuffling of the realizations of the selected sources (see Chávez et al. (2003); Lizier et al. (2011); Verdes (2005); Vicente et al. (2011) and the summary by Lizier (2014, Appendix A.5)), i.e., using a similar procedure to the one described in the *Maximum statistic test* section above. Testing all the selected sources collectively is in line with the perspective that the goal of the network inference is to find the *set* of relevant sources for each node.

#### Combining across multiple targets.

When the inference is performed on multiple targets, the omnibus *p* values can be employed in further statistical tests to control the family-wise error rate for the overall network (e.g., via FDR-correction; Benjamini & Hochberg, 1995; Dickhaus, 2014; which is implemented in the IDTxl toolbox).

It is important to fully understand the statistical questions and validation procedure implied by this approach. Combining the results across multiple targets by reusing the omnibus test *p* values for the FDR-correction yields a *hierarchical* test. The test answers two nested questions: (1) “*which nodes receive any significant overall information transfer?*” and, if any, (2) “*what is the structure of the incoming information transfer to each node?*.” However, the answers are computed in the reverse order, for the following reason: it would be computationally unfeasible to directly compute the collective transfer entropy from all candidate sources to the target right at the beginning of the network inference process. At this point, the candidate source set usually contains a large number of variables so that estimation will likely fall prey to the curse of dimensionality. Instead, a conservative *approximation* of the collective information transfer is obtained by considering only a subset of the potential sources, that is, those deemed significant by the maximum and minimum statistic tests described in the previous sections. Only if this approximation of the total information transfer is also deemed significant by the omnibus test (as well as by the FDR test at the network level), then the subset of significant sources for that target is interpreted post hoc as the local structure of the incoming information transfer. This way, the testing procedure exhibits a hierarchical structure: the omnibus test operates at the higher (global) level concerned with the collective information transfer, whereas the minimum and maximum tests operate at the lower (local) level of individual source-target connections.

Compared with a nonhierarchical analysis with a correction for multiple comparisons across all links (e.g., by network-wide Bonferroni correction or the use of the maximum statistic across all potential links), the above strategy buys both statistical sensitivity (“recall”) and the possibility to trivially parallelize computations across targets. The price to be paid is that a link with a relatively strong information transfer into a node with nonsignificant overall incoming information transfer may get pruned, while a link with relatively weaker information transfer into a node with significant overall incoming information transfer will prevail. This behavior clearly differs from a correction for multiple comparisons across all links. Arguably, this difference is irrelevant in many practical cases, although it could become noticeable for networks with high average in-degree and relatively uniform information transfer across the links. The difference can be reduced by setting a conservative critical threshold for the lower-level greedy analysis.

### Validation Tasks

For the purpose of the validation study, the additional assumptions of *causal sufficiency* and the *causal Markov condition* were made, such that the inferred network was expected to closely reflect the structural network for a sufficiently large sample size (Sun et al., 2015). Although this is not always the case, experiments under these conditions allow the evaluation of the performance of the algorithm with respect to an expected ground truth. An intuitive definition of these conditions is provided here, while the technical details are discussed at length in Spirtes et al. (1993). Moreover, the intrinsic stochastic nature of the processes makes purely synergistic and purely redundant interactions unlikely (and indeed vanishing for large sample size), thus satisfying the *faithfulness* condition (Spirtes et al., 1993).

The complete network inference algorithm implemented in the IDTxl toolkit (release v1.0) was validated on multiple synthetic datasets, where both the structural connectivity and the dynamics were known. Given the general scope of the toolkit, two dynamical models of broad applicability were chosen: a vector autoregressive process (VAR) and a coupled logistic maps process (CLM); both models are widely used in computational neuroscience (Rubinov et al., 2009; Valdes-Sosa et al., 2011; Zalesky et al., 2014), macroeconomics (Lorenz, 1993; Sims, 1980), and chaos theory (Strogatz, 2015).

The primary goal was to quantify the scaling of the performance with respect to the size of the network and the length of the time series. Sparse directed random Erdős-Rényi networks (Erdős & Rényi, 1959) of increasing size (*N* = 10 to 100 nodes) were generated with a link probability *p* = 3/*N* to obtain an expected in-degree of 3 links. Both the VAR and the CLM stochastic processes were repeatedly simulated on each causal network with increasingly longer time series (*T* = 100 to 10000 samples), a single replication (or trial, i.e., *R* = 1), and with 10 random initial conditions. The performance was evaluated in terms of precision, recall, and specificity in the classification of the links. Further simulations were carried out to investigate the influence of the critical alpha level for statistical significance and the performance of different estimators of conditional mutual information.

#### Vector autoregressive process.

The specific VAR process used in this study is described by the following discrete-time recurrence relation:

$$Y_{t}=\beta Y_{t-1}+\underset{X\in X_{Y}}{\sum }\alpha_{X}X_{t-l_{X}}+\eta_{t}$$ (7)

where ***X***_***Y***_ denotes the set of causal sources of the target process ***Y*** and a single random lag *l*_***X***_ ∈ {1, 2, 3, 4, 5} was used for each source ***X*** ∈ ***X***_***Y***_. A Gaussian noise term *η*_*t*_ with mean *μ* = 0 and standard deviation *θ* = 0.1 was added at each time step *t*; the noise terms added to different variables were uncorrelated. The self-coupling coefficient was set to *β* = 0.5 and the cross-coupling coefficients *α*_***X***_ were uniform and normalized for each target such that ∑_***X***∈***X***_***Y***__ *α*_***X***_ = 0.4. This choice of parameters guaranteed that the VAR processes were stable (the resulting spectral radii were between 0.9 and 0.95) and had stationary multivariate Gaussian distributions (Atay & Karabacak, 2006). As such, the Gaussian estimator implemented in IDTxl was employed for transfer entropy measurements in VAR processes. Note that transfer entropy and Granger causality (Granger, 1969) are equivalent for Gaussian variables (Barnett et al., 2009); therefore, using the Gaussian estimator with our algorithm can be viewed as extending Granger causality in the same multivariate/greedy fashion.

#### Coupled logistic maps process.

The coupled logistic maps process used in this study is described by the following discrete-time recurrence relations:

$$\begin{array}{ccc} a_{t} & = & \beta Y_{t-1}+\underset{X\in X_{Y}}{\sum }\alpha_{X}X_{t-l_{X}}\\ Y_{t} & = & (4a_{t}(1-a_{t})+\eta_{t})mod\;1 \end{array}$$ (8)

At each time step *t*, each node ***Y*** computes the weighted input *a*_*t*_ as a linear combination of its past value and the past of its sources, with the same conditions used for the VAR process on the choice of the random lags *l*_***X***_ and coupling coefficients *β* and *α*_***X***_. The value *Y*_*t*_ is then computed by applying the logistic map activation function *f*(*x*) = 4*x*(1 − *x*) to the weighted input *a*_*t*_ and adding the Gaussian noise *η*_*t*_ with the same properties used for the VAR process. Notice that the coefficient (*r* = 4) used in the logistic map function corresponds to the fully developed chaotic regime. The modulo-1 operation ensures that *Y*_*t*_ ∈ [0, 1] after the addition of noise. The *nearest-neighbor* estimators were employed for transfer entropy measurements in the analysis of the CLM processes (in particular, Kraskov’s estimator *I*^(1)^ with *k* = 4 nearest neighbors (Kraskov et al., 2004) and its extension to CMI Frenzel & Pompe, 2007; Gómez-Herrero et al., 2015; Vejmelka & Paluš, 2008). Nearest-neighbor estimators are model-free and are able to detect nonlinear dependencies in stochastic processes with non-Gaussian stationary distributions; fast CPU and GPU implementations are provided by the IDTxl package.

## RESULTS

### Influence of Network Size and Length of the Time Series

The aim of the first analysis was to quantify the scaling of the performance with respect to the size of the network and the length of the time series.

The inferred network was built by adding a directed link from a source node ***X*** to a target node ***Y*** whenever a significant transfer entropy from ***X*** to ***Y*** was measured while building the selected sources past set $X_{<t}^{S}$ (i.e., whenever ***X*** ∩ $X_{<t}^{S}$ ≠ ∅). The critical alpha level for statistical significance was set to *α*_max_ = 0.001 and *S* = 1000 surrogates were used for all experiments unless otherwise stated. The candidate sets for the target as well as the sources were initialized with a maximum lag of five (i.e., *l*_target_ = *l*_sources_ = 5, corresponding to the largest lag values used in the definition of the VAR and CLM processes).

The network inference performance was evaluated in comparison to the known underlying structural network as a binary classification task, using standard statistics based on the number of *true positives* (TP, i.e., correctly classified existing links), *false positives* (FP, i.e., absent links falsely classified as existing), *true negatives* (TN, i.e., correctly classified absent links), and *false negatives* (FN, i.e., existing links falsely classified as absent). The following standard statistics were employed in the evaluation:

**precision** = *TP*/(*TP* + *FP*)
**recall** = *TP*/(*TP* + *FN*)
**specificity** = *TN*/(*TN* + *FP*)

**Figure 3.**
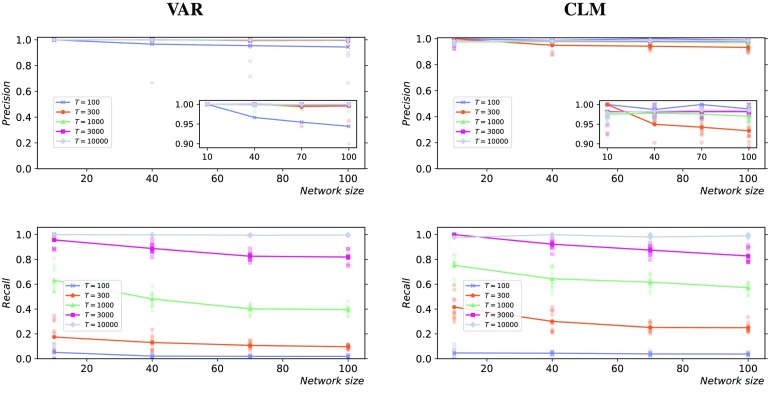
Precision (top) and recall (bottom) for different network sizes, sample sizes, and dynamics. Left: Vector autoregressive process; Right: Coupled logistic maps. Each subplot shows five curves, corresponding to different time series lengths (*T* = 100, 300, 1,000, 3,000, 10,000). The results for 10 simulations from different initial conditions are shown (low-opacity markers) in addition to the mean values (solid markers). All the random networks have an average in-degree *Np* = 3.

The plots in Figure 3 summarize the results in terms of precision and recall, while the specificity is additionally plotted in the Supporting Information. For both types of dynamics, the performance increased with the number of samples and decreased with the size of the network.

For shorter time series (*T* = 100 and *T* = 1000), the recall was the most affected performance measure as a function of *N* and *T*, while the precision and the specificity were always close to optimal (>98% on average). (Note that, while *S* = 1,000 is minimal for *α*_max_ = 0.001, recall was unchanged using *S* = 10,000 for *T* = 100.) For longer time series (*T* = 10,000), high performance according to all measures was achieved for both the VAR and CLM processes, regardless of the size of the network. The high precision and specificity are due to the effective control of the false positives, in accordance with the strict statistical significance level *α*_max_ = 0.001 (the influence of *α*_max_ is further discussed in the following sections). The inference algorithm was therefore conservative in the classification of the links.

### Validation of False Positive Rate

The critical alpha level for statistical significance *α*_max_ is a parameter of the algorithm that is designed to control the number of false positives in the network inference. As discussed in the *Statistical Tests* section in the Methods, *α*_max_ controls the probability that a target is a false positive, that is, that at least one of its sources is a false positive. This approach is in line with the perspective that the goal of the network inference is to find the *set* of relevant sources for each node.

**Figure 4.**
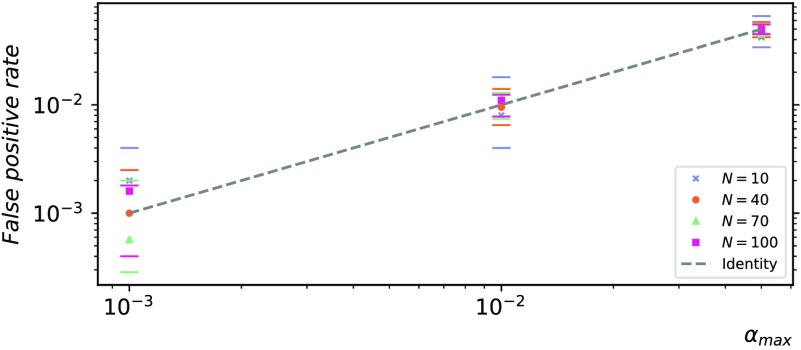
Validation of false positive rate for a single target (*t*_FPR_) on empty networks. The points indicate the average false positive rate over 50 simulations of a vector autoregressive process (*T* = 10,000). The horizontal marks indicate the corresponding 5th and 95th percentiles of the expected range. These were computed empirically from the distribution of the random variable 〈*X*_*j*_/*N*〉, where *X*_*j*_ ∼ *Binomial*(*N*, *α*_max_) are i.i.d. random variables, and the angular brackets indicate the finite average over 50 repetitions. The 5th percentile for *N* = 10 and *N* = 40 and *α*_max_ = 10^−3^ are equal to zero and therefore omitted from the log-log plot. The identity function is plotted as a reference (dashed line).

A validation study was carried out to verify that the final number of false positives is consistent with the desired level *α*_max_ after multiple statistical tests are performed. The *false positive rate* was computed after performing the inference on empty networks, where every inferred link is a false positive by definition (i.e., under the complete null hypothesis). The rate was in good accordance with the critical alpha threshold *α*_max_ for all network sizes, as shown in Figure 4.

The false positive rate validation was replicated in a scenario where the null hypothesis held for real fMRI data from the Human Connectome Project resting-state dataset (see Supporting Information). The findings are presented in the Supporting Information, together with a note on autocorrelation. Notably, the results on fMRI data are in agreement with the results on synthetic data shown in Figure 4.

### Influence of Critical Level for Statistical Significance

Given the conservative results obtained for both the VAR and CLM processes (Figure 3), a natural question is to what extent the recall could be improved by increasing the critical alpha level *α*_max_ and to what extent the precision would be negatively affected as a side effect.

**Figure 5.**
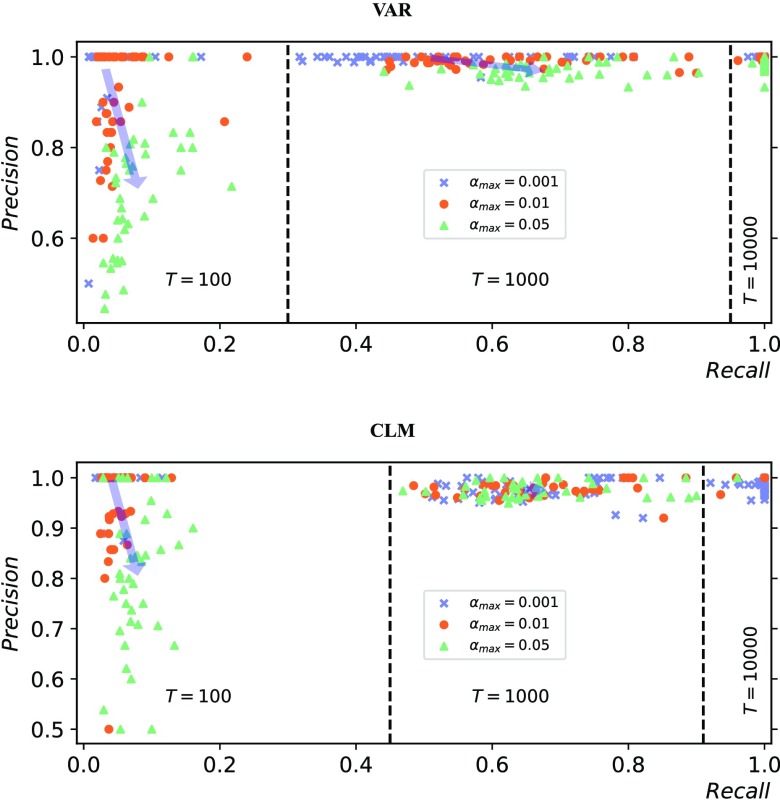
Influence of statistical significance threshold on network inference performance. Precision versus recall for different statistical significance levels (*α*_max_ = 0.05, 0.01, 0.001), corresponding to different colors. The plots summarize the results for different dynamics (Top: Vector autoregressive process; Bottom: Coupled logistic maps), different time series lengths (*T* = 100, 1,000, 10,000), and different network sizes (*N* = 10, 40, 70, 100, not distinguished). The arrows join the mean population values for the lowest and highest significance levels, illustrating the average trade-off between precision loss and recall gain.

In order to elucidate this trade-off, the analysis described above (Figure 3) was repeated for increasing values of *α*_max_, with results shown in Figure 5. For the shortest time series (*T* = 100), increasing *α*_max_ resulted in a higher recall and a lower precision, as expected; on the other hand, for the longest time series (*T* = 10,000), the performance measures were not significantly affected. Interestingly, for the intermediate case (*T* = 1,000), increasing *α*_max_ resulted in higher recall without negatively affecting the precision.

### Inference of Coupling Lags

So far, the performance evaluation focused on the identification the correct set of sources for each target node, regardless of the coupling lags. However, since the identification of the correct coupling lags is particularly relevant in neuroscience (see Wibral et al., 2013, and references therein), the performance of the algorithm in identifying the correct coupling lags was additionally investigated.

**Figure 6.**
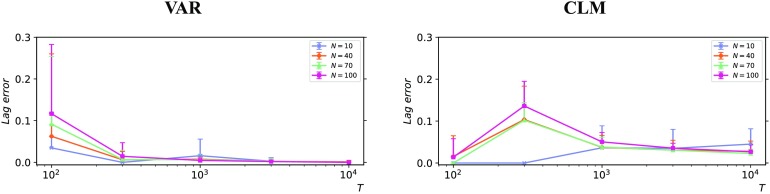
Average absolute error between the real and the inferred coupling lags, relative to the value expected at random. Results for different dynamics (Left: Vector autoregressive process; Right: Coupled logistic maps), different time series lengths (*T* = 100, 300, 1,000, 3,000, 10,000), and different network sizes (*N* = 10, 40, 70, 100). The error bars indicate the standard deviation over 10 simulations from different initial conditions.

By construction, a single coupling lag was imposed between each pair of processes (chosen at random between one and five discrete time steps, as described in the Methods). The average absolute error between the real and the inferred coupling lags was computed on the correctly recalled sources and divided by the value expected at random (which is the average absolute difference between two i.i.d. random integers in the [1, 5] interval). In line with the previous results on precision, the absolute error on coupling lag is consistently much smaller than that expected at random, even for the shortest time series (Figure 6). Furthermore, 1,000 samples were sufficient to achieve nearly optimal performance for both the VAR and the CLM processes, regardless of the size of the network. Note that as *T* increases and the recall increases, the lag error can increase (cf. *T* = 100 to 300 for the CLM process). This is perhaps because while the larger *T* permits more weakly contributing sources to be identified, it is not large enough to reduce the estimation error to make lag identification on these sources precise.

### Estimators

**Figure 7.**
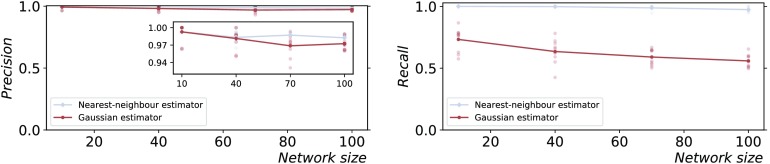
Gaussian versus nearest-neighbor estimator on the coupled logistic maps process. The precision (left) and recall (right) are plotted against the network size and a fixed time series length (*T* = 10,000 samples). The results for 10 simulations from different initial conditions are shown (low-opacity markers) in addition to the mean values (solid markers). The statistical significance level *α*_max_ = 0.05 was employed; an even larger gap between the recall of the estimators is obtained with *α*_max_ = 0.001.

Given its speed, the Gaussian estimator is often used for large datasets or as a first exploratory step, even when the stationary distribution cannot be assumed to be Gaussian. The availability of the ground truth allowed us to compare the performance of the Gaussian estimator and the nearest-neighbor estimator on the nonlinear CLM process, which does not satisfy the Gaussian assumption. As expected, the performance of the Gaussian estimator was lower than the performance of the nearest-neighbor estimator for all network sizes (Figure 7).

The hierarchical tests introduced in the Methods section allow running the network inference algorithm in parallel on a high-performance computing cluster. Such parallelization is especially needed when employing the nearest-neighbor estimator. In particular, each target node can be analyzed in parallel on a CPU (employing one or more cores) or a GPU, which is made possible by the CPU and GPU estimators provided by the IDTxl package (custom OpenCL kernels were written for the GPU implementation). A summary of the CPU and GPU run times is provided in the Supporting Information.

## DISCUSSION

The algorithm presented in this paper provides robust statistical tests for network inference to control the false positive rate. These tests are compatible with parallel computation on high-performance computing clusters, which enabled the validation study on synthetic sparse networks of increasing size (10 to 100 nodes), using different dynamics (linear autoregressive processes and nonlinear coupled logistic maps) and increasingly longer time series (100 to 10,000 samples). Both the network size and the sample size are one order of magnitude larger than previously demonstrated, showing feasibility for typical EEG and MEG experiments. The results demonstrate that the statistical tests achieve the desired false positive rate and successfully address the multiple-comparison problems inherent in network inference tasks (Figure 4).

The ability to control the false positives while building connectomes is a crucial prerequisite for the application of complex network measures, to the extent that Zalesky et al. (2016) concluded that “specificity is at least twice as important as sensitivity (i.e., recall) when estimating key properties of brain networks, including topological measures of network clustering, network efficiency and network modularity.” The reason is that false positives occur more prevalently between network modules than within them, and the spurious intermodular connections have a dramatic impact on network topology (Zalesky et al., 2016).

The trade-off between precision and recall when relaxing the statistical significance threshold was further investigated (Figure 5). When only 100 samples were used, the average recall gain was more than five times smaller than the average precision loss. In our opinion, this result is possibly due to the sparsity of the networks used in this study and suggests a conservative choice of the threshold for sparse networks and short time series. The trade-off was reversed for longer time series: when 1,000 samples were used, the average recall gain was more than five times larger than the average precision loss. Finally, for 10,000 samples, high precision and recall were achieved (>98% on average) for both the vector autoregressive and the coupled logistic maps processes, regardless of the statistical significance threshold.

For both types of dynamics, the network inference performance increased with the length of the time series and decreased with the size of the network (Figure 3). This is to be expected since larger systems require more statistical tests and hence stricter conditions to control the family-wise error rate (false positives). Specifically, larger networks result in wider null distributions of the maximum statistic (i.e., larger variance), whereas longer time series have the opposite effect. Therefore, for large networks and short time series, controlling the false positives can have a negative impact on the ability to identify the true positives, particularly when the effect size (i.e., the transfer entropy value) is small.

In addition, the superior ability of the nearest-neighbor estimator over the Gaussian estimator in detecting nonlinear dependencies was quantified. There is a critical motivation for this comparison: the general applicability of the nearest-neighbor estimators comes at the price of higher computational complexity and a significantly longer run time, so that the Gaussian estimator is often used for large datasets (or at least as a first exploratory step), even when the Gaussian hypothesis is not justified. To investigate such a scenario, the Gaussian estimator was tested on the nonlinear logistic map processes: while the resulting recall was significantly lower than the nearest-neighbor estimator for all network sizes, it was nonetheless able to identify over half of the links for a sufficiently large number (10,000) of time samples (Figure 7).

The stationarity assumption about the time series corresponds to assuming a single regime of neuronal activity in real brain recordings. If multiple regimes are recorded, which is typical in experimental settings (e.g., sequences of tasks or repeated presentation of stimuli interleaved with resting time windows), different stationary regimes can be studied by performing the analysis within each time window. The networks obtained in different time windows can either be studied separately and compared against each other or collectively interpreted as a single evolving temporal network. To obtain a sufficient amount of observations per window, multiple replications of the experiment under the same conditions are typically carried out. Replications can be assumed to be cyclo-stationary and estimation techniques exploiting this property have been proposed (Gómez-Herrero et al., 2015; Wollstadt et al., 2014); these estimators are also available in the IDTxl Python package. The convergence to the (unknown) causal network was only proven under the hypotheses of stationarity, causal sufficiency, and the causal Markov condition (Sun et al., 2015). However, conditional independence holds under milder assumptions (Runge, 2018) and the absence of links is valid under general conditions. The conditional independence relationships can, therefore, be used to exclude variables in following intervention-based causal experiments, making network inference methods valuable for exploratory studies.

In fact, the directed network is only one part of the model and provides the scaffold over which the information-theoretic measures are computed. Therefore, even if the structure of a system is known and there is no need for network inference, information theory can still provide nontrivial insights on the distributed computation by modeling the information storage, transfer, and modification within the system (Lizier, 2013). This decomposition of the predictive information into the active information storage and transfer entropy components is one out of many alternatives within the framework proposed by Chicharro & Ledberg (2012). Arguably, the storage-transfer decomposition reflects the segregation-integration dichotomy that has long characterized the interpretation of brain function (Sporns, 2010; Zeki & Shipp, 1988). Information theory has the potential to provide a quantitative definition of these fundamental but still unsettled concepts (Li et al., 2019). In addition, information theory provides a new way of testing fundamental computational theories in neuroscience, for example, predictive coding (Brodski-Guerniero et al., 2017).

As such, information-theoretic methods should not be seen as opposed to model-based approaches, but complementary to them (Friston et al., 2013). If certain physically motivated parametric models are assumed, the two approaches are equivalent for network inference: maximizing the log-likelihood is asymptotically equivalent to maximizing the transfer entropy (Barnett & Bossomaier, 2012; Cliff et al., 2018). Moreover, different approaches can be combined; for example, the recent large-scale application of spectral DCM was made possible by using functional connectivity models to place prior constraints on the parameter space (Razi et al., 2017). Networks inferred using bivariate transfer entropy have also been employed to reduce the model space prior to DCM analysis (Chan et al., 2017).

In conclusion, the continuous evolution and combination of methods show that network inference from time series is an active field of research and there is a current trend of larger validation studies, statistical significance improvements, and reduction of computational complexity. Information-theoretic approaches require efficient tools to employ nearest-neighbor estimators on large datasets of continuous-valued time series, which are ubiquitous in large-scale brain recordings (calcium imaging, EEG, MEG, fMRI). The algorithm presented in this paper is compatible with parallel computation on high-performance computing clusters, which enabled the study of synthetic nonlinear systems of 100 nodes and 10,000 samples. Both the network size and the sample size are one order of magnitude larger than previously demonstrated, bringing typical EEG and MEG experiments into scope for future information-theoretic network inference studies. Furthermore, the statistical tests presented in the Methods are generic and compatible with any underlying conditional mutual information or transfer entropy estimators, meaning that estimators applicable to spike trains (Spinney et al., 2017) can be used with this algorithm in future studies.

## ACKNOWLEDGMENTS

The authors acknowledge the Sydney Informatics Hub and the University of Sydney’s high-performance computing cluster Artemis for providing the high-performance computing resources that have contributed to the research results reported within this paper. Furthermore, the authors thank Aaron J. Gutknecht for commenting on a draft of this paper, and Oliver Cliff for useful discussions and comments.

## SUPPORTING INFORMATION

The network inference algorithm described in this paper is implemented in the open-source Python software package IDTxl (Wollstadt et al., 2019), which is freely available on GitHub (https://github.com/pwollstadt/IDTxl). In this paper, we refer to the current release (v1.0) at the time of writing (doi:10.5281/zenodo.2554339).

The raw data used for the experiment presented in the Supporting Information (https://doi.org/10.1162/netn_a_00092) is openly available on the MGH-USC Human Connectome Project database (https://ida.loni.usc.edu/login.jsp).

## ROLE INFORMATION

Leonardo Novelli: Conceptualization; Data Curation; Formal Analysis; Investigation; Software; Validation; Visualization; Writing - Original Draft; Writing - Review & Editing. Patricia Wollstadt: Conceptualization; Software; Writing - Review & Editing. Pedro Mediano: Software; Writing - Review & Editing. Michael Wibral: Conceptualization; Funding Acquisition; Methodology; Software; Supervision; Writing - Review & Editing. Joseph T. Lizier: Conceptualization; Funding Acquisition; Methodology; Software; Supervision; Writing - Review & Editing.

## FUNDING INFORMATION

Joseph T. Lizier, Universities Australia/German Academic Exchange Service (DAAD) Australia-Germany Joint Research Cooperation Scheme Grant: “Measuring Neural Information Synthesis and Its Impairment,” Award Id: 57216857. Michael Wibral, Universities Australia/German Academic Exchange Service (DAAD) Australia-Germany Joint Research Cooperation Scheme Grant: “Measuring Neural Information Synthesis and Its Impairment,” Award Id: 57216857. Joseph T. Lizier, Australian Research Council DECRA Grant, Award Id: DE160100630. Michael Wibral, Deutsche Forschungsgemeinschaft (DFG) Grant, Award Id: CRC 1193 C04. Joseph T. Lizier, Australian Research Council Discovery Grant, Award Id: DP160102742.

## TECHNICAL TERMS

IDTxl: The “Information Dynamics Toolkit xl” is an open-source Python package available on GitHub (see Supporting Information).
Markovian with finite memory: The present state of the target does not depend on the past values of the target and the sources beyond a maximum finite lag *l*_M_.
Nonuniform embedding: A set of nonuniformly spaced time lags that captures the underlying state of the process, akin to a Takens’ embedding.
Active information storage: The mutual information between the past and the present of the target: *I*($Y_{<t}^{S}$; *Y*_*t*_).
Step-down statistical test: A test which proceeds from the smallest to the largest *p* value. When the first non-significant *p* value is found, all the larger *p* values are also deemed not significant.
Causal sufficiency: The set of observed variables includes all their common causes (or the unobserved common causes have constant values).
Causal Markov condition: A variable X is independent of every other past variable conditional on all of its direct causes.
False positive rate: *FP*/(*FP* + *TN*).

## References

[bib1] Atay F. M., & Karabacak Ö. (2006). Stability of coupled map networks with delays. SIAM Journal on Applied Dynamical Systems, 5(3), 508–527.

[bib2] Ay N., & Polani D. (2008). Information flows in causal networks. Advances in Complex Systems, 11(01), 17–41.

[bib3] Barnett L., Barrett A. B., & Seth A. K. (2009). Granger causality and transfer entropy are equivalent for Gaussian variables. Physical Review Letters, 103(23), 238701. 20366183 10.1103/PhysRevLett.103.238701

[bib4] Barnett L., & Bossomaier T. (2012). Transfer entropy as a log-likelihood ratio. Physical Review Letters, 109(13), 138105. 23030125 10.1103/PhysRevLett.109.138105

[bib5] Benjamini Y., & Hochberg Y. (1995). Controlling the false discovery rate: a practical and powerful approach to multiple testing. Journal of the Royal Statistical Society. Series B (Methodological), 57(1), 289–300.

[bib6] Bossomaier T., Barnett L., Harré M., & Lizier J. T. (2016). An Introduction to Transfer Entropy. Springer International Publishing, Chambridge, UK.

[bib7] Brodski-Guerniero A., Paasch G.-F., Wollstadt P., Özdemir I., Lizier J. T., & Wibral M. (2017). Information-theoretic evidence for predictive coding in the face-processing system. The Journal of Neuroscience, 37(34), 8273–8283. 28751458 10.1523/JNEUROSCI.0614-17.2017 PMC6596791

[bib8] Chan J. S., Wibral M., Wollstadt P., Stawowsky C., Brandl M., Helbling S., … Kaiser J. (2017). Predictive coding over the lifespan: Increased reliance on perceptual priors in older adults—a magnetoencephalography and dynamic causal modelling study. bioRxiv Preprint, page 178095. 10.3389/fnagi.2021.631599 PMC8062739 33897405

[bib9] Chávez M., Martinerie J., & Le Van Quyen M. (2003). Statistical assessment of nonlinear causality: application to epileptic EEG signals. Journal of Neuroscience Methods, 124(2), 113–128. 12706841 10.1016/s0165-0270(02)00367-9

[bib10] Chicharro D., & Ledberg A. (2012). Framework to study dynamic dependencies in networks of interacting processes. Physical Review E, 86(4), 041901. 10.1103/PhysRevE.86.041901 23214609

[bib11] Cliff O., Prokopenko M., & Fitch R. (2018). Minimising the Kullback-Leibler divergence for model selection in distributed nonlinear systems. Entropy, 20(2), 51. 10.3390/e20020051 PMC7512642 33265171

[bib12] Cover T. M., & Thomas J. A. (2005). Elements of Information Theory. John Wiley & Sons, Hoboken, NJ, USA, 2 edition.

[bib13] Dickhaus T. (2014). Simultaneous Statistical Inference. Springer Berlin Heidelberg, Berlin, Heidelberg.

[bib14] Erdős P., & Rényi A. (1959). On random graphs. Publicationes Mathematicae Debrecen, 6, 290–297.

[bib15] Faes L., Nollo G., & Porta A. (2011). Information-based detection of nonlinear Granger causality in multivariate processes via a nonuniform embedding technique. Physical Review E, 83(5), 051112. 10.1103/PhysRevE.83.051112 21728495

[bib16] Frenzel S., & Pompe B. (2007). Partial mutual information for coupling analysis of multivariate time series. Physical Review Letters, 99(20), 204101. 18233144 10.1103/PhysRevLett.99.204101

[bib17] Friston K. J. (1994). Functional and effective connectivity in neuroimaging: a synthesis. Human Brain Mapping, 2(1–2), 56–78.

[bib18] Friston K. J., Moran R., & Seth A. K. (2013). Analysing connectivity with Granger causality and dynamic causal modelling. Current Opinion in Neurobiology, 23(2), 172–178. 23265964 10.1016/j.conb.2012.11.010 PMC3925802

[bib19] Garland J., James R. G., & Bradley E. (2016). Leveraging information storage to select forecast-optimal parameters for delay-coordinate reconstructions. Physical Review E, 93(2), 022221. 26986345 10.1103/PhysRevE.93.022221

[bib20] Gómez-Herrero G., Wu W., Rutanen K., Soriano M., Pipa G., & Vicente R. (2015). Assessing coupling dynamics from an ensemble of time series. Entropy, 17(4), 1958–1970.

[bib21] Granger C. W. J. (1969). Investigating causal relations by econometric models and cross-spectral methods. Econometrica, 37(3), 424–438.

[bib22] Kim P., Rogers J., Sun J., & Bollt E. M. (2016). Causation entropy identifies sparsity structure for parameter estimation of dynamic systems. Journal of Computational and Nonlinear Dynamics, 12(1), 011008.

[bib23] Kraskov A., Stögbauer H., & Grassberger P. (2004). Estimating mutual information. Physical Review E, 69(6), 066138. 10.1103/PhysRevE.69.066138 15244698

[bib24] Li M., Han Y., Aburn M. J., Breakspear M., Poldrack R. A., Shine J. M., & Lizier J. T. (2019). Transitions in brain-network level information processing dynamics are driven by alterations in neural gain. bioRxiv Preprint, page 581538. 10.1371/journal.pcbi.1006957 PMC6793849 31613882

[bib25] Lindner M., Vicente R., Priesemann V., & Wibral M. (2011). TRENTOOL: A Matlab open source toolbox to analyse information flow in time series data with transfer entropy. BMC Neuroscience, 12, 119. 22098775 10.1186/1471-2202-12-119 PMC3287134

[bib26] Lizier J. T. (2013). The Local Information Dynamics of Distributed Computation in Complex Systems. Springer Berlin, Heidelberg.

[bib27] Lizier J. T. (2014). JIDT: An information-theoretic toolkit for studying the dynamics of complex systems. Frontiers in robotics and AI, 1, 11.

[bib28] Lizier J. T., Heinzle J., Horstmann A., Haynes J.-D., & Prokopenko M. (2011). Multivariate information-theoretic measures reveal directed information structure and task relevant changes in fMRI connectivity. Journal of Computational Neuroscience, 30(1), 85–107. 20799057 10.1007/s10827-010-0271-2

[bib29] Lizier J. T., Prokopenko M., & Zomaya A. Y. (2008). Local information transfer as a spatiotemporal filter for complex systems. Physical Review E, 77(2), 026110. 10.1103/PhysRevE.77.026110 18352093

[bib30] Lizier J. T., Prokopenko M., & Zomaya A. Y. (2010). Information modification and particle collisions in distributed computation. Chaos, 20(3), 037109. 20887075 10.1063/1.3486801

[bib31] Lizier J. T., Prokopenko M., & Zomaya A. Y. (2012). Local measures of information storage in complex distributed computation. Information Sciences, 208, 39–54.

[bib32] Lizier J. T., & Rubinov M. (2012). Multivariate construction of effective computational networks from observational data. Technical Report Preprint 25/2012, Max Planck Institute for Mathematics in the Sciences.

[bib33] Lorenz H.-W. (1993). Chaotic dynamics in discrete-time economic models. In Nonlinear Dynamical Economics and Chaotic Motion (pages 119–166). Springer, Berlin.

[bib34] Montalto A., Faes L., & Marinazzo D. (2014). MuTE: A MATLAB toolbox to compare established and novel estimators of the multivariate transfer entropy. PLoS ONE, 9(10), e109462. 25314003 10.1371/journal.pone.0109462 PMC4196918

[bib35] Nichols T., & Hayasaka S. (2003). Controlling the familywise error rate in functional neuroimaging: a comparative review. Statistical Methods in Medical Research, 12(5), 419–446. 14599004 10.1191/0962280203sm341ra

[bib36] Paluš M., Albrecht V., & Dvoák I. (1993). Information theoretic test for nonlinearity in time series. Physics Letters A, 175(3–4), 203–209.

[bib37] Paninski L. (2003). Estimation of entropy and mutual information. Neural Computation, 15(6), 1191–1253.

[bib38] Razi A., Seghier M. L., Zhou Y., McColgan P., Zeidman P., Park H.-J., Sporns O., Rees G., & Friston K. J. (2017). Large-scale DCMs for resting-state fMRI. Network Neuroscience, 1(3), 222–241. 29400357 10.1162/NETN_a_00015 PMC5796644

[bib39] Roulston M. S. (1999). Estimating the errors on measured entropy and mutual information. Physica D: Nonlinear Phenomena, 125(3–4), 285–294.

[bib40] Rubinov M., Sporns O., van Leeuwen C., & Breakspear M. (2009). Symbiotic relationship between brain structure and dynamics. BMC Neuroscience, 10, 55. 19486538 10.1186/1471-2202-10-55 PMC2700812

[bib41] Runge J. (2018). Causal network reconstruction from time series: from theoretical assumptions to practical estimation. Chaos, 28(7), 075310. 30070533 10.1063/1.5025050

[bib42] Runge J., Heitzig J., Petoukhov V., & Kurths J. (2012). Escaping the curse of dimensionality in estimating multivariate transfer entropy. Physical Review Letters, 108(25), 258701. 23004667 10.1103/PhysRevLett.108.258701

[bib43] Runge J., Nowack P., Kretschmer M., Flaxman S., & Sejdinovic D. (2018). Detecting causal associations in large nonlinear time series datasets. arXiv Preprint. arXiv: 1702.07007. 10.1126/sciadv.aau4996 PMC6881151 31807692

[bib44] Schreiber T. (2000). Measuring information transfer. Physical Review Letters, 85(2), 461–464. 10991308 10.1103/PhysRevLett.85.461

[bib45] Schreiber T., & Schmitz A. (2000). Surrogate time series. Physica D: Nonlinear Phenomena, 142(3–4), 346–382.

[bib46] Shannon C. E. (1948). A mathematical theory of communication. Bell System Technical Journal, 27(3), 379–423.

[bib47] Šidák Z. (1967). Rectangular confidence regions for the means of multivariate normal distributions. Journal of the American Statistical Association, 62(318), 626–633.

[bib48] Sims C. A. (1980). Macroeconomics and reality. Econometrica, 48(1), 1–48.

[bib49] Spinney R. E., Prokopenko M., & Lizier J. T. (2017). Transfer entropy in continuous time, with applications to jump and neural spiking processes. Physical Review E, 95(3), 032319. 28415203 10.1103/PhysRevE.95.032319

[bib50] Spirtes P., Glymour C., & Scheines R. (1993). Causation, Prediction, and Search, volume 81 of *Lecture Notes in Statistics* Springer New York.

[bib51] Sporns O. (2010). Networks of the Brain. MIT Press, Cambridge, MA.

[bib52] Strogatz S. H. (2015). Nonlinear Dynamics and Chaos. CRC Press, Boca Raton, FL.

[bib53] Sun J., Taylor D., & Bollt E. M. (2015). Causal network inference by optimal causation entropy. SIAM Journal on Applied Dynamical Systems, 14(1), 73–106.

[bib54] Takens F. (1981). Detecting strange attractors in turbulence. In Rand D. and Young L., editors, Dynamical Systems and Turbulence, pages 366–381. Springer, Berlin.

[bib55] Vakorin V. A., Krakovska O. A., & McIntosh A. R. (2009). Confounding effects of indirect connections on causality estimation. Journal of Neuroscience Methods, 184(1), 152–160. 19628006 10.1016/j.jneumeth.2009.07.014

[bib56] Valdes-Sosa P. A., Roebroeck A., Daunizeau J., & Friston K. J. (2011). Effective connectivity: influence, causality and biophysical modeling. NeuroImage, 58(2), 339–361. 21477655 10.1016/j.neuroimage.2011.03.058 PMC3167373

[bib57] Vejmelka M., & Paluš M. (2008). Inferring the directionality of coupling with conditional mutual information. Physical Review E, 77(2), 026214. 10.1103/PhysRevE.77.026214 18352110

[bib58] Verdes P. F. (2005). Assessing causality from multivariate time series. Physical Review E, 72(2), 026222. 10.1103/PhysRevE.72.026222 16196699

[bib59] Vicente R., Wibral M., Lindner M., & Pipa G. (2011). Transfer entropy—a model-free measure of effective connectivity for the neurosciences. Journal of Computational Neuroscience, 30(1), 45–67. 20706781 10.1007/s10827-010-0262-3 PMC3040354

[bib60] Vlachos I., & Kugiumtzis D. (2010). Nonuniform state-space reconstruction and coupling detection. Physical Review E, 82(1), 016207. 10.1103/PhysRevE.82.016207 20866707

[bib61] Wibral M., Pampu N., Priesemann V., Siebenhühner F., Seiwert H., Lindner M., … Vicente R. (2013). Measuring information-transfer delays. PLoS ONE, 8(2), e55809. 23468850 10.1371/journal.pone.0055809 PMC3585400

[bib62] Wollstadt P., Lizier J. T., Vicente R., Finn C., Martínez-Zarzuela M., Mediano P., … Wibral M. (2019). IDTxl: The Information Dynamics Toolkit xl: a Python package for the efficient analysis of multivariate information dynamics in networks. Journal of Open Source Software, 4(34), 1081.

[bib63] Wollstadt P., Martínez-Zarzuela M., Vicente R., Díaz-Pernas F. J., & Wibral M. (2014). Efficient transfer entropy analysis of non-stationary neural time series. PLoS ONE, 9(7), e102833. 25068489 10.1371/journal.pone.0102833 PMC4113280

[bib64] Zalesky A., Fornito A., Cocchi L., Gollo L. L., & Breakspear M. (2014). Time-resolved resting-state brain networks. Proceedings of the National Academy of Sciences, 111(28), 10341–10346. 10.1073/pnas.1400181111 PMC4104861 24982140

[bib65] Zalesky A., Fornito A., Cocchi L., Gollo L. L., van den Heuvel M. P., & Breakspear M. (2016). Connectome sensitivity or specificity: which is more important? NeuroImage, 142, 407–420. 27364472 10.1016/j.neuroimage.2016.06.035

[bib66] Zeki S., & Shipp S. (1988). The functional logic of cortical connections. Nature, 335, 311–317. 3047584 10.1038/335311a0

