# Supplementary material for: Large-scale directed network inference with multivariate transfer entropy and hierarchical statistical testing
Source: Netw Neurosci. 2019 Jul 1;3(3):827–47. doi: 10.1162/netn_a_00092 (PMC6663300; doi:10.1162/netn_a_00092)
Supplement: Supplementary file 1 [file netn-03-827-s001.pdf]

Novelli, L., Wollstadt, P., Mediano, P., Wibral, M., & Lizier, J.T. (2019). Supporting information for "Large-scale directed network inference with multivariate transfer entropy and hierarchical statistical testing." *Network Neuroscience*, *3*(3), 827–847. https://doi.org/10.1162/netn_a_00092

SUPPORTING INFORMATION

Run-time on CPU and GPU

The number of transfer entropy calculations scales as $\mathcal{O}(N^2 d l_{\text{max}} S)$, where N is the number of processes, d is the average inferred in-degree, l_{max} is the maximum temporal search depth per process (i.e., $l_{\text{max}} = max\{l_{\text{target}}, l_{\text{sources}}\}$), and S is the number of surrogates. This assumes that d is independent of the network size N; however, note that $d = N$ in the worst case of a fully connected network, leading to cubic run-times.

The network inference algorithm was designed for parallelisation:

- When using the CPU estimators, it is possible to parallelise over targets, resulting in $\mathcal{O}(N d l_{\text{max}} S)$ transfer entropy calculations per target for the nearest-neighbour estimator and $\mathcal{O}(N d l_{\text{max}})$ transfer entropy calculations for the Gaussian estimator (if using analytic null distributions instead of surrogates). The complexity of each calculation is $\mathcal{O}(kTlogT)$ for the nearest-neighbour estimator and $\mathcal{O}(T)$ for the Gaussian estimator (where T is number of time series samples and k is the number of nearest-neighbours).
- When using the GPU estimators, it is possible to parallelise both over targets and surrogates. Each target requires $\mathcal{O}(N d l_{\text{max}})$ transfer entropy calculations including surrogates, assuming that all surrogates for a source fit into the GPU's main memory and can be processed in parallel. The complexity of each calculation is $\mathcal{O}(T^2)$. If enough memory is available, it is further possible to parallelise over time samples T, resulting in faster run-times in practice.

The practical run-time for a full network analysis that considers each process as a target depends on the number of available computing nodes. In the worst case, where only a single computing node is available, the full run-time is equal to the single-target run-time multiplied by N, since the target are analysed in series. In the best case, if N computing nodes are available, the full run-time is equal to the single-target run-time, since all targets can be analysed in parallel. Notice that there is a trade-off between run-time and memory requirements: if all the targets are analysed in parallel, the full required memory in N times larger than the memory required in the single-node case; conversely, if the targets are analysed in series, the full required memory is equal to the memory required in the single-node case.

In the experiments presented in this article, the algorithm was either run using a *single core* per target (on different Intel Xeon CPUs with similar characteristics: 2.1-2.6 GHz), or using a whole dedicated GPU per target (NVIDIA V100 SXM2, 16 GB RAM). These computations were performed on the Artemis computing cluster made available by the Sydney Informatics Hub at The University of Sydney. The maximum CPU and GPU run-times for a single target are shown in Table 2, which summarises the results for different time series lengths and different network sizes. Notice that the CPU run-time per target can be reduced if multiple cores per target are available.

Sample size<br>T	Network size<br>N	max GPU time per target (h)	max CPU time per target (h)	max CPU memory per target (GB)
100	10	0.005	0.01	<0.50
	40	0.01	0.05	<0.50
	70	0.02	0.10	<0.50
	100	0.03	0.15	0.63
1000	10	0.05	0.63	0.65
	40	0.20	2.30	0.70
	70	0.25	4.30	0.70
	100	0.45	5.50	0.70
10 000	10	0.85	13.00	5.50
	40	3.30	120.00	6.60
	70	5.00	250.00	7.40
	100	6.70	335.00	8.00

Table 2. Maximum CPU and GPU run-time for a single target using the nearest-neighbour estimator and 200 surrogates. Summary of the results for different time series lengths ($T = 100$, 1000, 10 000) and different network sizes ($N = 10, 40, 70, 100$).

Validation of false positive rate on real fMRI data

The false positive rate validation (presented in Figure 4 for synthetic VAR data) was replicated in a scenario where the null hypothesis held for real data. Once again, the aim was to verify that the false positive rate was consistent with the desired level $\alpha_{\max}$. The Human Connectome Project resting state fMRI dataset (Van Essen et al., 2012) was used for this purpose (see Supporting Information). The raw data was pre-processed by applying a 3rd order Butterworth bandpass filter (0.01-0.08 Hz), then cutting 200 samples from the start and the end of the time series to remove potential filtering artefacts (leaving 800 samples for the analysis). In order to build a scenario where the null hypothesis held, 10 different random regions of interest (ROIs) were selected from different random subjects, such that the corresponding time series were expected to be independent of each other. The network inference was performed with the same settings used in the null test on synthetic data but employing the nearest-neighbour estimator, since the real data could not be assumed to follow a Gaussian distribution. The results on fMRI data are presented in Figure 8 and are consistent with the previous results on synthetic data (Figure 4).

Unless appropriate measures are taken, the strong autocorrelation typically found in real data would result in an inflated false positive rate for short time series (an effect already observed by Barnett and Seth (2011) when using Granger causality). The issue is addressed in IDTxl by means of the *dynamic correlation exclusion*, also known as *Theiler window* (Kantz & Schreiber, 2003; Theiler, 1986), as originally suggested for transfer entropy estimation by Schreiber (2000). The idea is to exclude the closest points in time from the nearest-neighbour search which is necessary for the estimation of the transfer entropy (when using nearest-neighbour estimators). The autocorrelation decay time (i.e., the shortest time shift such that the autocorrelation function drops by a factor of $1/e$ with respect to the zero-shift value

(Lindner et al., 2011)) is used as a heuristic to adapt the size of the Theiler window to the data.

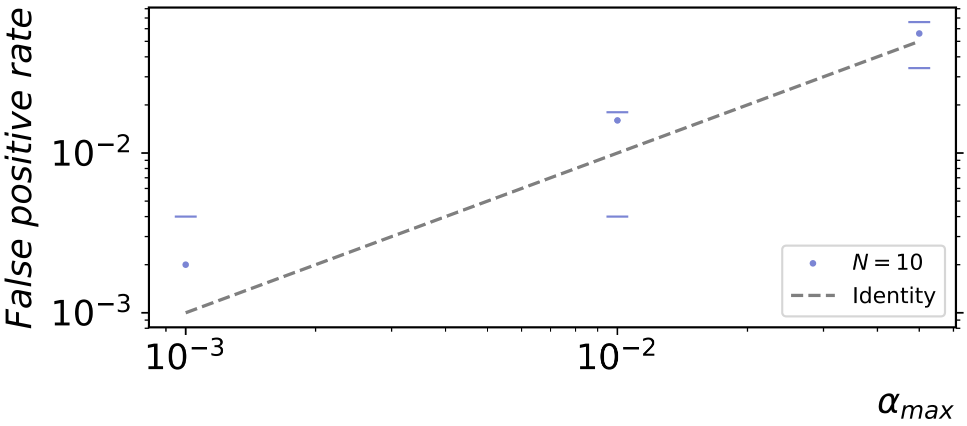

Figure 8. Validation of false positive rate for a single target (t_{FPR}) on real fMRI data. The points indicate the average false positive rate over 50 repetitions of the experiment using random regions ($N = 10$) from different subjects. The horizontal marks indicate the corresponding 5th and 95th percentiles of the expected range. These were computed empirically from the distribution of the random variable $\langle X_j/N \rangle$, where $X_j \sim \mathit{Binomial}(N, \alpha_{\mathrm{max}})$ are i.i.d. random variables, and the angular brackets indicate the finite average over 50 repetitions. The identity function is plotted as a reference (dashed line).

Alternative visualisations of performance scaling by network and sample size

Figure 9 replots the precision and recall from Figure 3 using different subplots for each sample size, and additionally shows the specificity. Similarly, Figure 10 replots the precision and recall from Figure 5 using different subplots for each sample size.

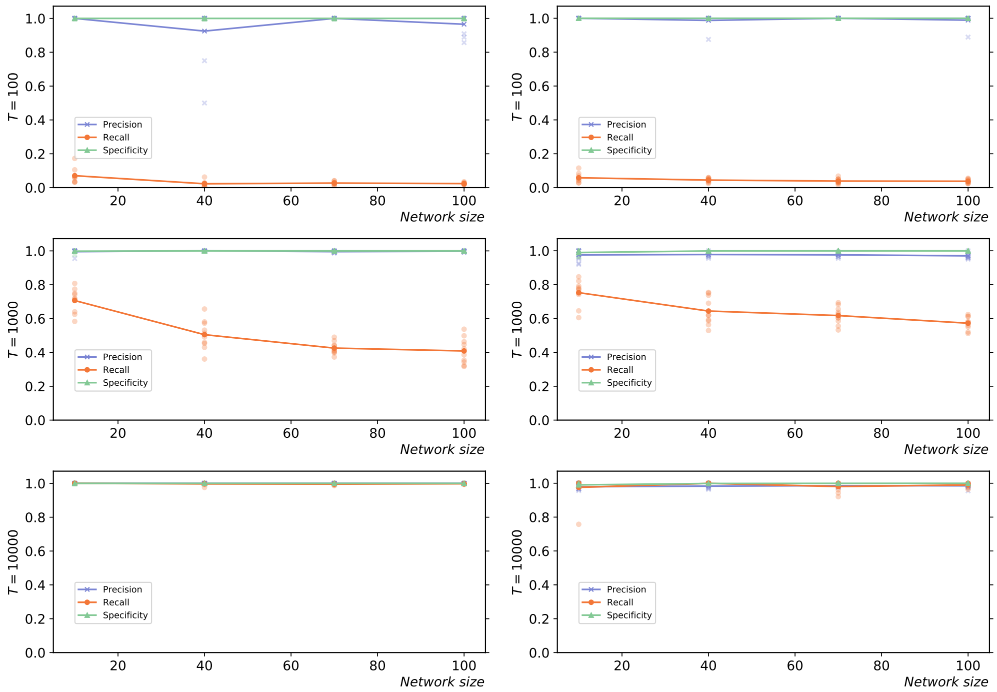

Figure 9. Precision, recall, and specificity for different network sizes, sample sizes, and dynamics. Left: Vector autoregressive process; Right: Coupled logistic maps. Each row corresponds to a different time series length (Top: $T = 100$; middle: $T = 1000$, bottom: $T = 10\,000$). The results for 10 simulations from different initial conditions are shown (low-opacity circles) in addition to the mean values (solid circles).

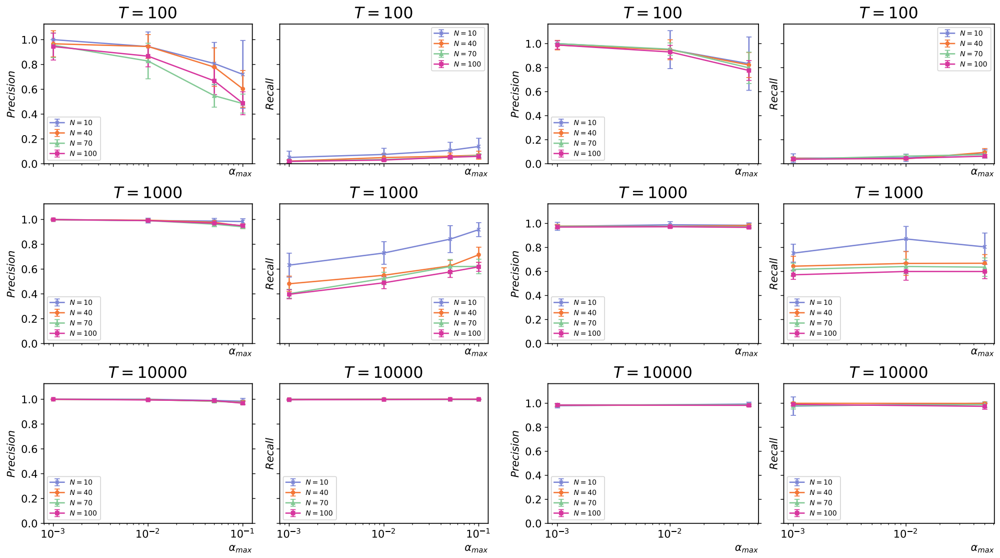

Figure 10. Precision-recall trade-off for different statistical significance levels. The plots show the results for different dynamics (Left: Vector autoregressive process; Right: Coupled logistic maps), different time series lengths (top to bottom: $T = 100$, 1000, 10 000), and different network sizes ($N = 10$, 40, 70, 100). The error bars indicate the standard deviation over 10 simulations from different initial conditions.

REFERENCES

Barnett, L., & Seth, A. K. (2011). Behaviour of Granger causality under filtering: Theoretical invariance and practical application. *Journal of Neuroscience Methods*, *201*(2), 404–419. doi: 10.1016/j.jneumeth.2011.08.010

Kantz, H., & Schreiber, T. (2003). *Nonlinear time series analysis* (2nd ed.). Cambridge University Press. doi: 10.1017/CBO9780511755798

Lindner, M., Vicente, R., Priesemann, V., & Wibral, M. (2011). TRENTOOL: A Matlab open source toolbox to analyse information flow in time series data with transfer entropy. *BMC Neuroscience*, *12*, 119. doi: 10.1186/1471-2202-12-119

Schreiber, T. (2000). Measuring Information Transfer. *Physical Review Letters*, *85*(2), 461–464. doi: 10.1103/PhysRevLett.85.461

Theiler, J. (1986). Spurious dimension from correlation algorithms applied to limited time-series data. *Physical Review A*, *34*(3), 2427–2432. doi: 10.1103/PhysRevA.34.2427

Van Essen, D., Ugurbil, K., Auerbach, E., Barch, D., Behrens, T., Bucholz, R., . . . Yacoub, E. (2012). The Human Connectome Project: A data acquisition perspective. *NeuroImage*, *62*(4), 2222–2231. doi: 10.1016/j.neuroimage.2012.02.018
